# Menstrual hygiene practice and associated factors among adolescent girls in sub-Saharan Africa: a systematic review and meta-analysis

**DOI:** 10.1186/s12889-022-14942-8

**Published:** 2023-01-06

**Authors:** Etsay Woldu Anbesu, Dejen Kahsay Asgedom

**Affiliations:** Department of Public Health, College of Medical and Health Sciences, Samara University, Samara, Ethiopia

**Keywords:** Pooled prevalence, Determinants, Menstrual hygiene practice, Adolescent girls, Systematic review, Meta-analysis, Sub-Saharan Africa

## Abstract

**Background:**

Menstrual hygiene has not received adequate attention in Sub-Saharan Africa, and there is a lack of regional representative data. Therefore, this study aimed to estimate the pooled prevalence of good menstrual hygiene practices and associated factors among adolescent girls in sub-Saharan Africa.

**Methods:**

In this study**,** the Preferred Reporting Items for Systematic Reviews and Meta-Analysis guidelines were used to develop the review manuscript. Online electronic databases, such as PubMed/Medline, Google Scholar, and CINAHL, were searched to retrieve available studies. The database search was conducted from January 1 to May 17, 2022. The selection, quality assessment, and data extraction of the studies were performed. Quality assessment of the studies was performed using the Joanna Briggs Institute Meta-Analysis of Statistics Assessment and Review Instrument. Subgroup analysis and meta-regression were performed based on country, study area, and sample size. Publication bias was examined by funnel plots and Egger’s test. The statistical analysis was conducted using STATA version 14 software and RevMan software, and statistical significance was declared at a *p* value of less than 0.05.

**Protocol registration number:**

CRD42020165628.

**Results:**

A total of 229 studies were retrieved, and 14 studies were included in the final meta-analysis. The pooled prevalence of good menstrual hygiene practices was 45% (95% CI, (37, 53). Adolescents from urban residences (OR = 3.03, 95% CI (2.3, 3.97)), able to afford menstrual sanitary products (OR = 2.17, 95% CI (1.42, 3.3)), and from educated mothers (OR = 2.33, 95% CI (1.32, 4.12)) were associated with increased odds of good menstrual hygiene practice.

**Conclusion:**

The pooled prevalence of menstrual hygiene practices was low compared to the SDG 6.2 target by 2030. “Achieve access to adequate and equitable sanitation and hygiene for all, paying special attention to the needs of women and girls and those in vulnerable situations”. Therefore, improving the accessibility of a safe water supply, hygiene, sanitation facilities and affordability of menstrual products and promoting maternal education are mandatory and should be part of government-level public health policy to prevent related health issues, loss of economic output and education opportunities.

**Supplementary Information:**

The online version contains supplementary material available at 10.1186/s12889-022-14942-8.

## Introduction

Menstruation is a physiological process that occurs among girls and women [1]. Menstrual hygiene management (MHM) practice is described as using clean menstrual management material, washing the body as needed with soap and water, and having access to facilities to dispose of used materials [2].

Worldwide, inappropriate management of menstruation affects girls and women in developing countries [3, 4]. In developing countries, approximately 12.3% to 75% of girls cannot access or afford clean sanitary materials, and they use low-quality products such as new or old clothes, cotton wool, toilet paper, underwear alone, and sponges [5–10]. According to a United Nations International Children Emergency Fund (UNICEF) report, 10% of school-age African girls do not attend school during menstruation [11]. A study performed in five sub-Saharan African countries showed that the majority of adolescent girls reported a lack of safe, private, clean toilets and washing facilities at schools [9].

A review of the literature showed that factors associated with poor menstrual hygiene practice were lack of access to clean and effective absorbents, facilities to change, disposal of absorbents, soap, water, and privacy [3, 5, 12, 13]. In addition, consideration of menstruation as a taboo leads to fear and shame in discussions with other families and reduces young girls’ knowledge about menstrual hygiene practices. Even adult women may not be aware of the biological factors of good hygienic practices and may reduce their menstrual hygiene practices [14–16].

Poor menstrual hygiene practices have many consequences, including exposing adolescent girls and women to reproductive organ and urogenital infections, psychosocial stress, and reduced opportunities for accessing school and jobs [13, 17–19]. Studies have revealed that many school girls suffer from concentration and limited participation during class times due to discomfort and dishonor during menstruation [20–22].

In Sub-Saharan African countries, menstruation among school-age girls and women is a neglected issue. Adequate attention was not given by the water, sanitation, and hygiene (WASH), education sectors, or sexual and reproductive health programs, despite the formal inclusion of menstrual hygiene under reproductive health [23], and there are no regional representative data. Thus, this systematic review and meta-analysis aimed to estimate the pooled prevalence of menstrual hygiene practice and identify its associated factors among adolescent girls in sub-Saharan Africa.

## Methods

### Study protocol registration and reporting

The Preferred Reporting Items for Systematic review and Meta-analysis (PRISMA) guidelines were used to develop the systematic review and meta-analysis [24]. The PRISMA-P 2009 checklist was used to report the search process. The protocol was registered at PROSPERO with registration number CRD42020165628.

### CoCoPop/PEO search guide

Condition: menstrual hygiene practice Context: sub-Saharan Africa *Population*: adolescent girl (10–19 years) [25]. *Exposure*: exposure is a determinant that increases or decreases the likelihood of menstrual hygiene practice among adolescent girls in sub-Saharan Africa. These factors include but are not limited to residence, age, maternal educational level, family income, menstrual flow duration, and knowledge of menses. Outcome measurement: The primary outcome of the study was the pooled prevalence of good menstrual hygiene practices. Good menstrual hygiene practices were indicated when the studies reported overall good menstrual hygiene practices for the different measurements of menstrual hygiene practices (type of menstrual items used, maintenance of items if reusable or disposal if one-time use only, changing frequency, using clean menstrual management material to absorb or collect, washing the body as needed with soap, access to water, and disposal sites). The secondary outcome of the study was to identify determinates of menstrual hygiene practice among adolescent girls. Event and control data were extracted from the original studies in the Microsoft Excel sheet and analysed using RevMan software. The criteria for selecting independent variables were how consistently and frequently they were reported in the primary studies. Accordingly, determinants reported in more than one study and having consistent classification were included.

## Search strategy

The search strategies were performed using the Preferred Reporting Items for Systematic Reviews and Meta-Analysis guidelines. Online electronic databases such as PubMed/Medline, Google Scholar, CINAHL, and African Journal Online were used to search articles. The database search was conducted from January 1 to May 17, 2022. As Google Scholar generally retrieved a high number of articles whenever searched, for each of the search terms, we retrieved the first 10 pages. The retrieved studies were exported to Endnote version 8 reference manager software [26]. The procedure for the search and selection of studies was reported using the PRISMA diagram*.* A cross-reference search was performed to add other studies from the final included studies. Medical Subject Heading (MeSH) terms and entry terms were searched from MeSH term databases. The search string was developed using Boolean operators (OR, AND), and modifications were made depending on the specific requirements of the database (Additional file 1).

## Study selection and eligibility criteria

The two authors (EW and DK) independently screened the studies based on the inclusion and exclusion criteria. Duplicate, irrelevant titles and abstracts were removed. The relevant articles with the full text were further screened for quality appraisal. In the case of articles not open access, we contacted the corresponding author, and the articles were excluded for not responding authors. During the review of the studies, any disagreement among reviewers was resolved by further discussion. This study included all observational studies: cross-sectional, analytical cross-sectional, case–control, and cohort studies. Articles published only in English and studies that reported overall good menstrual hygiene practices and their associated factors were included. Moreover, a study that only reported overall good menstrual hygiene practices was included. Both institution- and community-based studies were included. We considered only quantitative results for studies that examined both quantitative and qualitative results. Case reports, conference reports, reviews and expert opinions were excluded. Restriction was not made to the date of study publication.

## Quality assessments

Articles were assessed using their title, abstract, and a full text review before the inclusion of articles in the final meta-analysis. A critical appraisal was performed by two authors (EW and DK) using the Joanna Briggs Institute Meta-Analysis of Statistics Assessment and Review Instrument (JBI-MASt-ARI) [27] (Additional file 2). Studies with a quality scale of 50% and above were included and considered for systematic review and meta-analysis. For any scoring disagreements between the authors, the sources of discrepancy were resolved with discussion.

## Data extraction and management

After identifying all eligible articles, two independent reviewers (EW and DK) extracted the relevant data using an organized format on Microsoft Excel Spreadsheet 2016. Pretesting the data extraction form was performed before the beginning of the actual data extraction. In case variations of extracted data existed, the phase was repeated, and then discrepancies between data extractors were resolved by discussion. For the final included articles, we extracted the author name, year of publication, study area, study design, study period, sample size, response rate prevalence, study subjects, events and control data of the outcome variable. For any unclear data that might be encountered during data extraction, communication was made with the corresponding authors of the primary studies.

## Data synthesis and analysis

The extracted data were imported into STATA version 14 software and extracted to RevMan software. Tables and figures were used to summarize random effects, and a narrative description of the included studies was performed. A random-effect model was used to estimate the overall pooled prevalence of menstrual hygiene practice and its determinants [28]. The existence of an association between the factors and menstrual hygiene practice was estimated based on the effect size, and the statistical significance level was declared at a *p* value of less than 0.05. We assessed heterogeneity by using the I^2^ statistic test [29]. I^2^ values of 25%, 50%, and 75% were representative of low, moderate, and significant heterogeneity, respectively. Subgroup analysis and meta-regression were performed based on country and study area (community/school) characteristics to investigate sources of heterogeneity.

Sensitivity analysis was performed to determine the effects of the studies on the overall estimation, and publication bias was examined by the visual inspection of funnel plots [30] and Egger’s test [31]. A *p* value < 0.05 on Egger’s test was considered indicative of statistically significant publication bias.

## Results

### Study selection

**Fig. 1:**
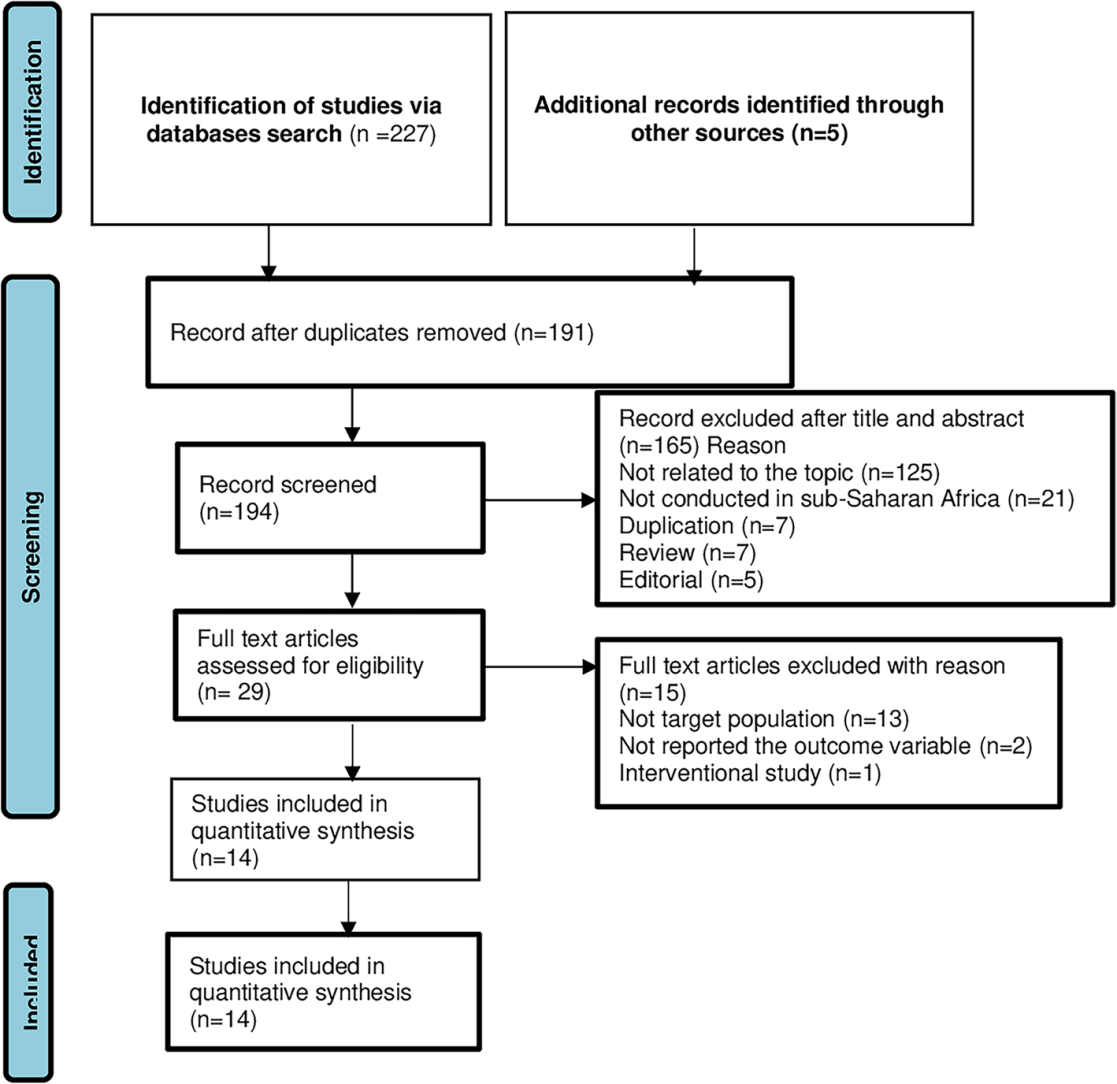
Flow chart of the study selection for meta-analysis of menstrual hygiene practice among adolescent girls in Sub-Saharan Africa, 2022

A total of 229 studies were retrieved from the online electronic databases PubMed/Medline (*n* = 54), Google Scholar (*n* = 160), CINAHL (*n* = 6), and African Journal Online (*n* = 12). The titles, abstracts, and full texts of the studies were screened. Thirty-eight duplicated articles were removed, and 165 articles with irrelevant titles and abstracts were excluded for not related to the topic (*n* = 125), not conducted in sub-Saharan Africa (*n* = 21), duplication (*n* = 7), review (*n* = 5), and editorial (*n* = 5). Twenty-six full-text articles were assessed for quality appraisals, and 15 articles were excluded for not targeting the age group (*n* = 12), being an interventional study (*n* = 1) and not reporting the outcome variable (*n* = 2). Three articles were identified through a cross-reference search of the included studies, and two articles were identified from websites. Finally, 14 articles were included in the final systematic review and meta-analysis (Fig. 1).

### Quality appraisal

All included studies satisfied four out of eight JBI critical assessments (at least 50%). The requirements for inclusion were specified in fourteen studies. All included studies employed suitable statistical methods and confounding factor management strategies. Nevertheless, because all of the included studies were cross-sectional, this study was not able to identify confounding factors (Table 1). Moreover, studies were excluded during quality appraisal based on the inclusion criteria (Supplementary file Table 3).

**Table 1:** Quality appraisal of included studies based on the Joanna Briggs Institute (JBI) critical appraisal tool

Studies	Q1	Q2	Q3	Q4	Q5	Q6	Q7	Q8	TOTAL	Study subjects
Azage, et al. 2018 [32]	No	Yes	Yes	Yes	Not applicable	Yes	Yes	Yes	Yes = 6 No = 1 Not applicable = 1	15–19 years adolescent girls
Bulto. 2021 [33]	No	Yes	Yes	Yes	Not applicable	Yes	Yes	Yes	Yes = 6 No = 1 Not applicable = 1	13–19 years adolescent girls
Mohammed G. 2020 [34]	No	Yes	Yes	Yes	Not applicable	Yes	Yes	Yes	Yes = 6 No = 1 Not applicable = 1	10–19 years adolescent girls
Boakye-Yiadom, et al. 2018 [35]	Yes	Yes	Yes	Yes	Not applicable	Yes	Yes	Yes	Yes = 7 Not applicable = 1	10–19 years adolescent girls
Mohammed, S. 2020 [36]	No	Yes	Yes	Yes	Not applicable	Yes	Yes	Yes	Yes = 6 No = 1 Not applicable = 1	10–19 years adolescent girls
Fehintola, et al. 2017 [37]	No	Yes	Yes	Yes	Not applicable	Yes	Yes	No	Yes = 5 No = 2 Not applicable = 1	10–19 years adolescent girls
Belayneh, et al. 2019 [38]	No	Yes	Yes	Yes	Not applicable	Yes	Yes	Yes	Yes = 6 No = 1 Not applicable = 1	10–19 years adolescent girls
Anchebi H,et al. 2017 [39]	Yes	Yes	Yes	Yes	Not applicable	Yes	Yes	Yes	Yes = 6 Not applicable = 1	14–19 years adolescent girls
Serbesa ML, et al. 2018 [40]	Yes	Yes	Yes	Yes	Not applicable	Yes	Yes	No	Yes = 6 No = 1 Not applicable = 1	13–19 years adolescent girls
Kumbeni MT, et al. 2020 [41]	Yes	Yes	Yes	Yes	Not applicable	Yes	Yes	No	Yes = 6 No = 1 Not applicable = 1	10–19 years adolescent girls
Fisseha MA, et al. 2017 [6]	No	Yes	Yes	Yes	Not applicable	Yes	Yes	Yes	Yes = 6 No = 1 Not applicable = 1	14–19 years adolescent girls
Upashe, S.P., et al. 2015 [42]	Yes	Yes	Yes	Yes	Not applicable	Yes	Yes	Yes	Yes = 6 No = 1 Not applicable = 1	10–19 years adolescent girls
Nnennaya, Esther Umahi, et al [43]	Yes	Yes	Yes	Yes	Not applicable	Yes	Yes	No	Yes = 6 No = 1 Not applicable = 1	10–19 years adolescent girls
Habtegiorgis, Yohannes, et al [44]	Yes	Yes	Yes	Yes	Not applicable	Yes	Yes	Yes	Yes = 7 Not applicable = 1	13–19 adolescent girls

### Characteristics of the included studies

A total of 14 cross-sectional studies with 7416 participants were included in the final systematic review and meta-analysis. The studies were published from 2015 to 2021 in different sub-Saharan African countries. Of the 14 studies, 9 studies were in Ethiopia [6, 32–34, 38–40, 42, 44], 3 studies were in Ghana [35, 36, 41], and 2 studies were in Nigeria [37, 43]. The sample size of the included studies ranged from 250 in Ghana [36] to 1,006 in Ethiopia [32]. The prevalence of good menstrual hygiene practices ranges from 24.4% to 66.8% in Ethiopia [32, 40]. Moreover, 14 studies performed an analysis to identify factors associated with menstrual hygiene practice in Sub-Saharan Africa (Table 2).

**Table 2:** Summary characteristics of studies included in the meta-analysis of menstrual hygiene practice in Sub-Saharan Africa, 2022

Author/s/year/(ref)	Study area	Country	Study design	Sample size	Response rate (%)	Prevalence (%)	Study subjects	Factors associated with menstrual hygiene practice
Azage, et al. 2018 [32]	Schools in Bahir dar city	Ethiopia	Cross sectional	1006	99.6	24.4	15–19 years adolescent girls	Being older, attending formal education, educational status of participants’ mother
Bulto. 2021 [33]	Preparatory and high schools in Holeta Town	Ethiopia	Cross sectional	403	97.1	34.7	13–19 years adolescent girls	adolescents from urban residence (AOR = 2.62, 95% CI: 1.53–4.48), got information about menstruation from mothers (AOR = 2.17, 95% CI: 1.18–3.96) and teachers (AOR = 5.09, 95% CI: 2.67–9.67), school toilets with inside lock (AOR = 2.82, 95% CI: 1.67–4.76), not missing school during menstruation (AOR = 4.2, 95% CI: 1.55–11.41), experienced menstrual-related problems (AOR = 2.63, 95% CI: 1.49–4.64), experienced any whitish or gray discharge per-vagina (AOR = 2.84, 95% CI: 1.66–4.85), having good overall knowledge about menstruation (AOR = 1.94, 95% CI: 1.07–3.52)
Mohammed G. 2020 [34]	Secondary School in East Hararghe Zone	Ethiopia	Cross sectional	672	99.4	58.3	10–19	living in urban areas (AOR = 2.59, 95% CI: 1.77, 3.80), having moderate (AOR = 2.78, 95% CI: 1.64, 5.28), good knowledge about menstruation (AOR = 3.87, 95% CI: 2.21, 6.77), mothers’ secondary and above education (AOR = 1.83, 95% CI: 1.01, 3.30)
Boakye-Yiadom, et al. 2018 [45]	Schools in the Yendi Municipality	Ghana	Cross sectional	386	93.6	31.1	10–19 years adolescent girls	access to funds, having adequate knowledge of menstruation
Mohammed, S. 2020 [46]	Kumbungu in the Northern Region of Ghana	Ghana	Cross sectional	250	100	50.8	10–19 years adolescent girls	Aged, fathers occupation, receive regular allowance for menstrual care, fear of staining clothing, fear of being teased, no availability of sanitary pad, lack of private place to manage period at school
Fehintola, et al. 2017 [37]	selected public schools in Ogbomoso	Nigeria	Cross sectional	447	100	25.3	10–19 years adolescent girls	Having an educated mother, living with their
Belayneh, et al. 2019 [38]	Gedeo zone high school	Ethiopia	Cross sectional	791	98.14	39.7	10–19 years adolescent girls	Age less than 15 years [OR = 1.71:95% CI (1.22, 2.39)], longer days of menstrual flow [OR = 2.51:95% CI (1.66, 3.80)], poor knowledge of menses [OR = 1.48:95% CI (1.04, 2.1)]
Anchebi H,et al. 2017 [39]	High schools in Adama town	Ethiopia	Cross sectional	398	94.3	57.03	14–19 years adolescent girls	Mothers’ education status [AOR = 0.608; 95% CI = 0.374–0.990], source of money for sanitary materials [AOR = 2.267; 95% CI = 1.076, 4.772], respondents feeling on comfortability of the school [AOR = 0.557; 95% CI = 0.366–0.846]
Serbesa ML, et al. 2018 [40]	Batu high school	Ethiopia	Cross sectional	274	100	66.7	13–19 years adolescent girls	Residence, parents' educational status, religion, family monthly income, types of sanitary materials
Kumbeni MT, et al. 2020 [47]	schools in Talensi district	Ghana	Cross sectional	705	97	61.4	10–19 years adolescent girls	Mothers' education, parents' socioeconomic
Fisseha MA, et al. 2017 [6]	schools in Wegera district	Ethiopia	Cross sectional	423	100	29.8	14–19 years adolescent girls	Exposure to advertisements on sanitary napkins (AOR 2.06(1.27, 3.34)), good knowledge of menstrual hygiene (AOR 2.23(1.06, 4.71))
Upashe, S.P., et al. 2015 [42]	schools in Nekemte town	Ethiopia	Cross sectional	828	98	39.8	10–19 years adolescent girls	Good knowledge of menstruation (AOR = 1.51, 95% CI = 1.02 – 2.22), having radio/TV (AOR = 2.42, 95% CI: 1.64 – 3.56), educational status of the mother (AOR = 2.03, 95% CI = 1.38 – 2.97), earning permanent pocket money from parents (AOR = 2.73, 95% CI = 1.76 – 4.26)
Nnennaya, Esther Umahi, et al. 2021 [43]	schools in Jalingo town	Nigeria	Cross sectional	297	100	57.6	10–19 years adolescent girls	Good knowledge of menstrual hygiene management
Habtegiorgis, Yohannes, et al.[44]	High school in Dessie city	Ethiopia	Cross sectional	536	98.2	53.9	13–19 adolescent girls	Age range 16–19 years (AOR = 1.93, 95% CI: [1.22–3.06]); school grade level 10 (AOR = 1.90, 95% CI: [1.18–3.07]); maternal education (primary) (AOR = 3.72, 95% CI: [1.81–7.63]), maternal education (secondary) (AOR = 8.54, 95% CI: [4.18–17.44]), maternal education (college) (AOR = 6.78, 95% CI: [3.28–14.02]), respectively]; having regullar menses [AOR = 1.85, 95% CI: (1.03–3.32); good knowledge regarding menstruation (AOR = 2.02, 95% CI: [1.32–3.09]); discussing menstrual hygiene with friends (AOR = 1.79, 95% CI: [1.12–2.86]), and obtaining money for pads from the family (AOR = 2.08, 95% CI: [1.15–3.78])

### The pooled prevalence of menstrual hygiene practices in Sub-Saharan Africa

**Fig. 2:**
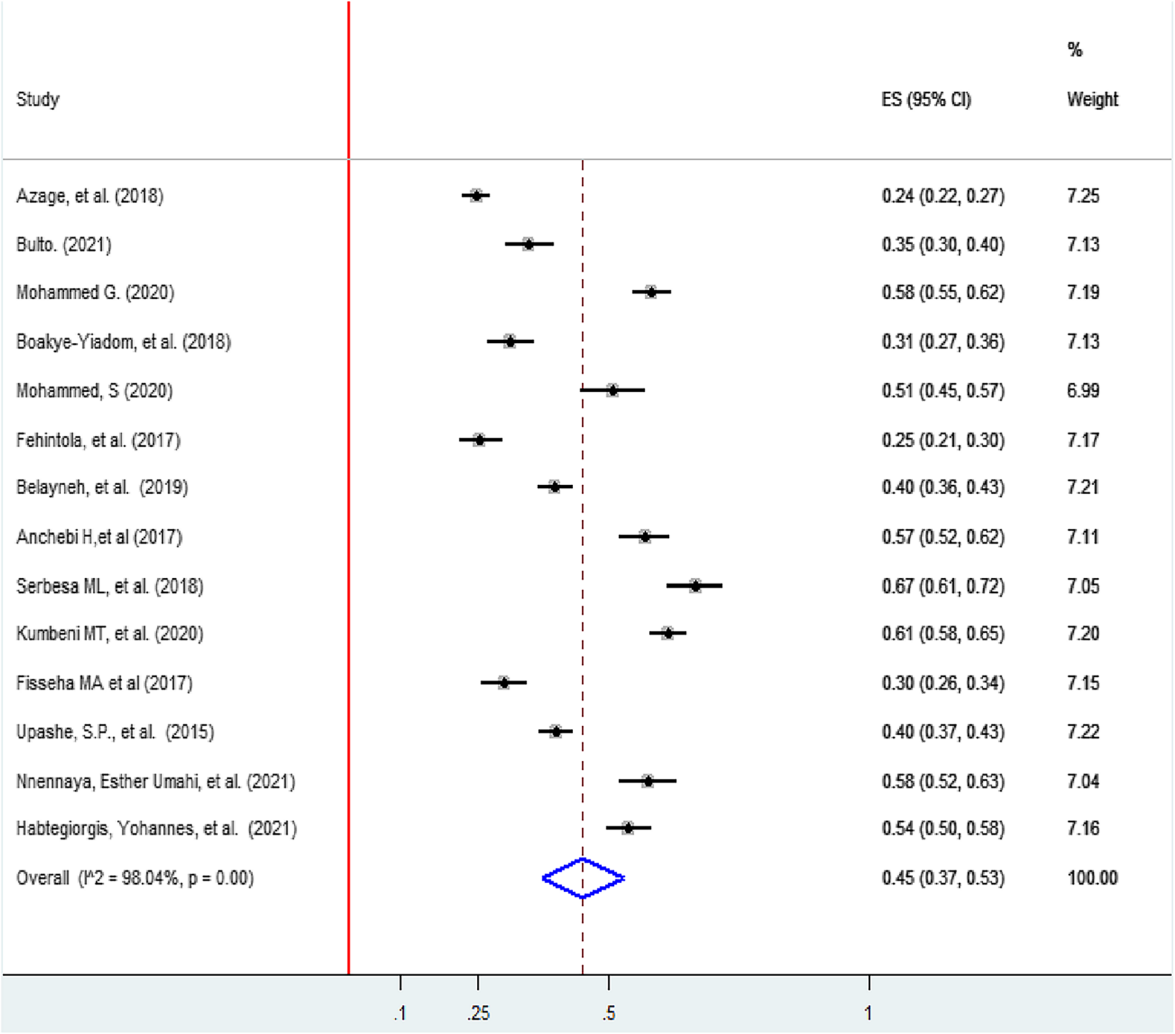
Forest plot showing the pooled prevalence of good menstrual hygiene among adolescent girls in Sab-Saharan Africa, 2022

The pooled prevalence of good menstrual hygiene practice in Sub-Saharan Africa was 45% (95% CI, (37, 53), with significant heterogeneity between studies (I2 = 98.4%, *p* ≤ 0.001). The proportion of included studies ranged from 24% (95%, CI: 22, 27) [32] to 67% (95%, CI: 61, 72) [40] in Ethiopia (Fig. 2).

### Subgroup analysis

Subgroup analysis was performed by country and study area characteristics, and studies performed in Ethiopia and school-based studies showed the source of heterogeneity (I^2^ ≥ 97.64, *p* ≤ 0.001). The pooled prevalence of good menstrual hygiene practices based on country was 45% in Ethiopia and 48% in Ghana (Table 3).

**Table 3:** Subgroup analysis for the prevalence of menstrual hygiene practice in Ethiopia, 2022

Sub group	Number of included studies	Prevalence (95% CI)	Heterogeneity statistics	
			*P* value	I^2^
By country				
Ethiopia	9	45 (35, 54)	< 0.001	98.12%
Ghana	3	48(28, 67)	< 0.001	0.00%
Nigeria	2	36(33, 40)	< 0.001	0.00%
By Study area				
Community based	3	45(22, 68)	< 0.001	0.00%
School based	11	45(37, 53)	< 0.001	97.64%

### Meta regression

Meta-regression was performed to identify the source of heterogeneity for the pooled prevalence of good menstrual hygiene practices. Country, study area and sample size were considered, and none of them showed the source of heterogeneity (*p* value > 0.05) (Additional file 3: Table S1)*.*

### Publication bias

The presence of publication bias was checked using funnel plots, and visual inspection of the funnel plot suggested symmetry, as seven studies were on the right side and seven studies were on the left side (Additional file 4: Figure S1). Moreover, publication bias was not shown by Egger’s test (*p* = 0.754) (Additional file 5: Table S2*).*

### Determinants of menstrual hygiene practice in sub-Saharan Africa

In this study, 8 studies [6, 32–34, 36, 38, 44, 48] were included in the analysis of determinants of menstrual hygiene practice. Five factors were assessed for meta-analysis, and high heterogeneity was observed for age, maternal education, and knowledge factors. Determinants that were assessed for menstrual hygiene practice among adolescent girls in sub-Saharan Africa included two articles on residence (1,075 participants), [33, 34], five articles on age (3,006 participants) [6, 32, 36, 38, 44], three articles on maternal educational status (2,214 participants) [32, 34, 44], five articles on knowledge status (2,825 participants), [6, 33, 34, 38, 44], and three articles on the affordability of menstrual equipment (1,614 participants), [36, 44, 48].

**Fig. 3:**
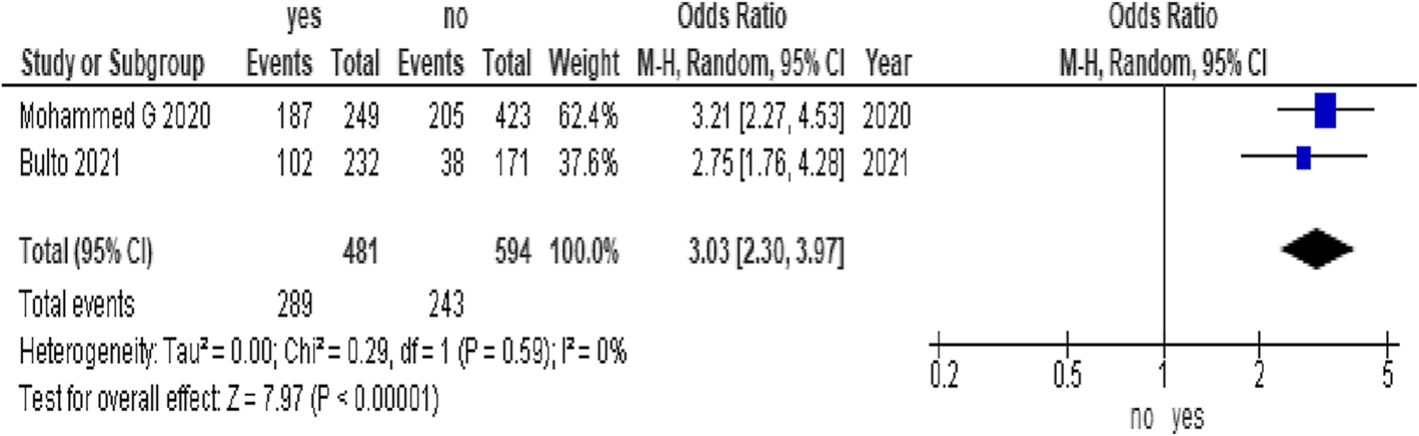
Forest plot showing the association between residence and menstrual hygiene practice in Sab-Saharan Africa

**Fig. 4:**
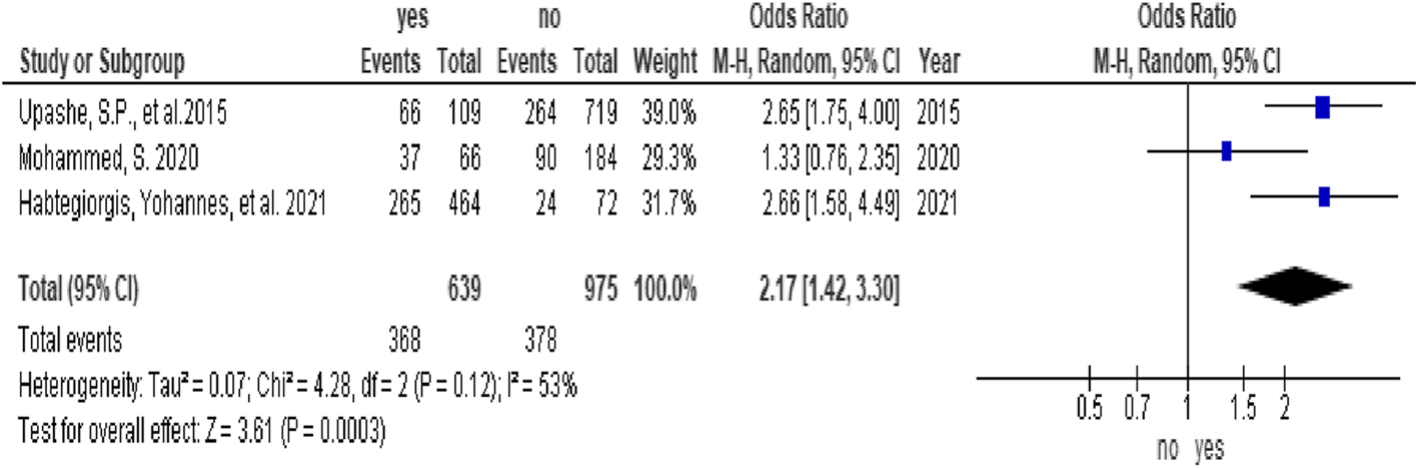
Forest plot showing the association between the affordability and menstrual hygiene practice in Sab-Saharan Africa

**Fig. 5:**
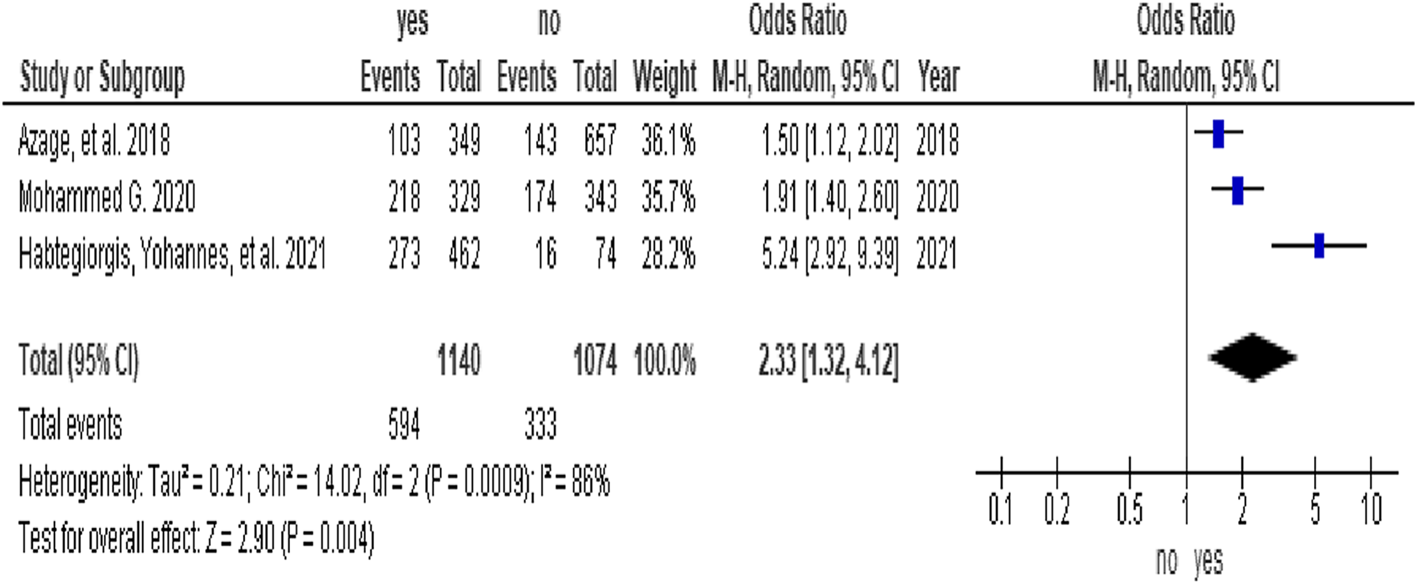
Forest plot showing the association between the maternal education and menstrual hygiene practice in Sab-Saharan Africa

**Fig. 6:**
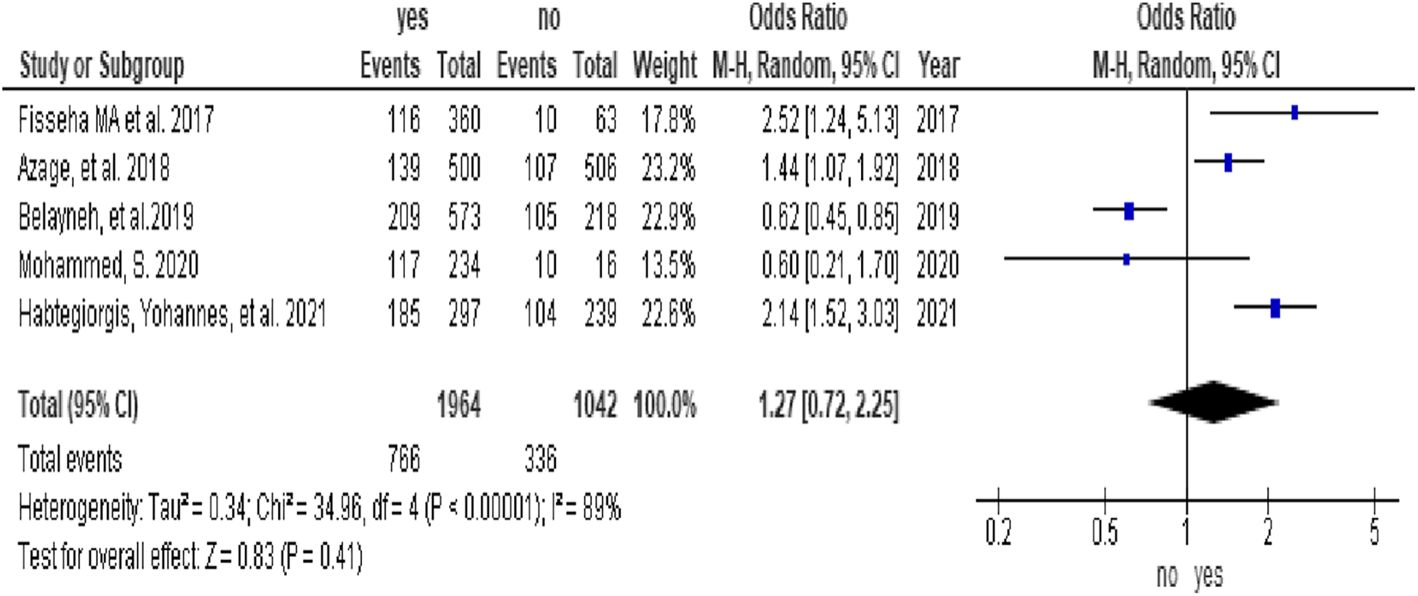
Forest plot showing the the association between age and menstrual hygiene practice in Sab-Saharan Africa

**Fig. 7:**
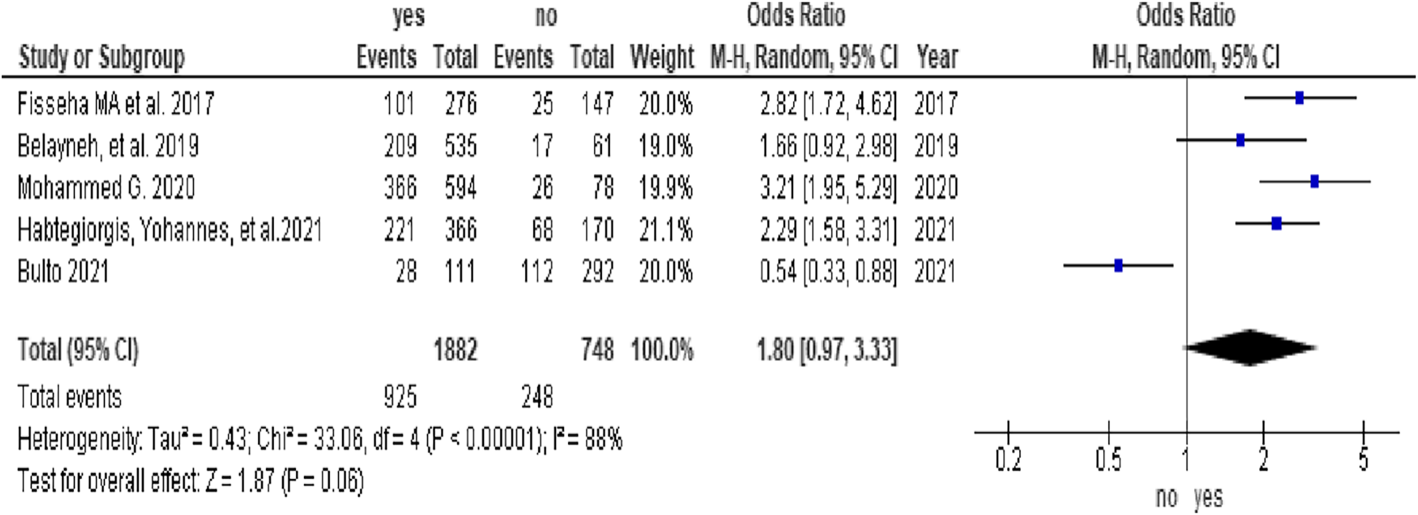
Forest plot showing the association between knowledge and menstrual hygiene practice in Sub-Sahara Africa

In this study, adolescent girls from urban settings who were able to afford menstrual sanitary products and from educated mothers were associated with good menstrual hygiene practices. The odds of menstrual hygiene practice among adolescent girls from urban settings were 3.03 times higher than those among adolescent girls from rural settings (OR = 3.03, 95% CI (2.3, 3.97)) (Fig. 3). The odds of menstrual hygiene practice among adolescent girls who were able to afford menstrual sanitary products were 2.17 times higher than those among adolescent girls who were not able to afford menstrual sanitary products (OR = 2.17, 95% CI (1.42, 3.3)) (Fig. 4). The odds of menstrual hygiene practice among adolescent girls from educated mothers were 2.33 times higher than those among their counterparts (OR = 2.33, 95% CI (1.32, 4.12)) (Fig. 5). In this study, the age and knowledge of the participants were statistically insignificant (Fig. 6 and Fig. 7, respectively).

## Discussion

This systematic review and meta-analysis aimed to assess the pooled prevalence of good menstrual hygiene practice and its associated factors in sub-Saharan Africa. Poor menstrual hygiene practices affect the health of millions of adolescent girls in developing countries [49, 50]. To the best of our knowledge, no systematic review or meta-analysis has been conducted on the pooled prevalence of good menstrual hygiene practices and their associated factors in Sub-Saharan Africa. Moreover, there are inconsistent findings on menstrual hygiene practice in sub-Saharan Africa. Therefore, this systematic review and meta-analysis will help policy-makers, programmers, planners, clinicians, and researchers design appropriate strategies.

In this study, the pooled prevalence of good menstrual hygiene practice among adolescent girls was 45% (95% CI (37, 53)). This finding was in line with studies conducted in India, 45% [51], Nepal, 40% [52], and Lao PDR, 44% [53]. However, the pooled prevalence was lower than that in the Dang District of Nepal, 67.0% [54]. The difference might be due to differences in sample size, study setting, study period, availability, and access to health services.

The odds of menstrual hygiene practice among adolescent girls from urban settings were 3.03 times higher than those among adolescent girls from rural settings (OR = 3.03, 95% CI (2.3, 3.97)). This was consistent with studies performed in India [55] and low-income countries [8]. It might be the fact that girls in rural areas of developing countries have limited awareness of menstrual hygiene management and face substantial challenges in different settings [56]. Moreover, there is a lack of accessibility and affordability to sanitary products, functional latrines, safe water supply, hygiene, and sanitation facilities in rural areas [21, 57–61].

The odds of menstrual hygiene practice among adolescent girls who were able to afford menstrual sanitary products were 2.17 times higher than those among adolescent girls who were not able to afford menstrual sanitary products (OR = 2.17, 95% CI (1.42, 3.3)). This was consistent with studies performed in India [62]. This might be because girls who have access to money from their parents can buy sanitary napkins. It has been noted that girls from low-income families lack access to menstrual hygiene facilities, and the cost of sanitary products is one of the main barriers to good menstrual hygiene practices during menstruation [62].

The odds of menstrual hygiene practice among adolescent girls from educated mothers were 2.33 times higher than those among their counterparts (OR = 2.33, 95% CI (1.32, 4.12)). This was consistent with other studies [63, 64]. This might be because as societal taboos and stigma challenge traditional social norms, mothers are the main source of information on menstrual hygiene for adolescents [65]. Moreover, adolescent girls in developing countries often grow up with limited knowledge of menstruation and low male involvement to support menstrual hygiene management for their wives and daughters [9, 15, 66].

This study has the following limitations. Heterogeneity and articles published in languages other than English were not considered. In this review, the outcome variable might be affected by other confounding variables, as all studies were cross-sectional. In this study, only three countries (Ethiopia, Ghana and Nigeria) were included, representing only a small proportion of sub-Saharan African countries. Additional database searches, such as Scopus and EMBASE, were not performed due to the lack of free access, and we recommend funding to expand database searches. Relevant studies may be missed, as we retrieved the first 10 pages in Google Scholar. Despite these limitations, we performed a comprehensive search of studies from databases, gray literature, and cross-referencing of included studies to include all relevant studies.

## Conclusion

The pooled prevalence of menstrual hygiene practice was low compared to the SDG 6.2 target by 2030. “Achieve access to adequate and equitable sanitation and hygiene for all, paying special attention to the needs of women and girls and those in vulnerable situations”. Therefore, improving the accessibility of a safe water supply, hygiene, sanitation facilities and affordability of menstrual products and promoting maternal education are mandatory and should be part of government-level public health policy to prevent related health issues, loss of economic output and education opportunities.

## Supplementary Information

**Additional file 1. **Database search MeSH terms and entry terms

**Additional file 2. **JBI critical appraisal checklist for analytical cross-sectional studies

**Additional file 3: Table S1.** Meta-regression analysis of factors with menstrual hygiene practice in Sub-Saharan Africa, 2022

**Additional file 4: Figure S1 **Funnel plots to test the publication bias of the 14 studies, 2022

**Additional file 5: Table S2. **Publication bias using Egger’s test on menstrual hygiene among adolescents in sub Saharan Africa, 2022

**Additional file 6: Supplementary file Table 3.** Quality appraisal of excluded studies

## Data Availability

All data generated or analysed during the current study are included in this manuscript and its supplementary information files. Any further requests for data are available with the corresponding author.

## Abbreviations

PROSPERO: International Prospective Register of systematic Reviews
CRD: Clinical data repository
CINAHL: Cumulative Index to Nursing and Allied health literature
CI: Confidence interval
OR: Odds ratio
MHM: Menstrual hygiene management
UNICEF: United Nations International Children Emergency Fund
WASH: Water, sanitation, and hygiene
PRISMA: The Preferred Reporting Items for Systematic review and Meta-analysis
STATA: Statistical software for data science
MeSH: Medical Subject Heading
JBI-MASt-ARI: Joanna Briggs Institute Meta-Analysis of Statistics Assessment and Review Instrument; I^2^: square statistics
SRH: Sexual and reproductive health
