# Supplementary material for: Menstrual hygiene practice and associated factors among adolescent girls in sub-Saharan Africa: a systematic review and meta-analysis
Source: BMC Public Health. 2023 Jan 6;23:33. doi: 10.1186/s12889-022-14942-8 (PMC9817285; doi:10.1186/s12889-022-14942-8)
Supplement: Supplementary file 1 — Additional file 1. Database search MeSH terms and entry terms [file 12889_2022_14942_MOESM1_ESM.docx]

Additional file 1: Database search MeSH terms and entry terms

PubMed/Medline

((((((((((((((((((((((((((((((((Menstrual Hygiene Products[MeSH Terms]) OR (Menstrual Hygiene Products[Title/Abstract])) OR (Hygiene Product, Menstrual[Title/Abstract])) OR (Hygiene Products, Menstrual[Title/Abstract])) OR (Menstrual Hygiene Product[Title/Abstract])) OR (Menstrual Products[Title/Abstract])) OR (Menstrual Product[Title/Abstract])) OR (Menstrual Cup[Title/Abstract])) OR (Cup, Menstrual[Title/Abstract])) OR (Cups, Menstrual[Title/Abstract])) OR (Menstrual Cups[Title/Abstract])) OR (Tampons, Menstrual[Title/Abstract])) OR (Tampon, Menstrual[Title/Abstract])) OR (Menstrual Tampons[Title/Abstract])) OR (Vaginal Tampon[Title/Abstract])) OR (Tampon, Vaginal[Title/Abstract])) OR (Tampons, Vaginal[Title/Abstract])) OR (Vaginal Tampons[Title/Abstract])) OR (Menstrual Tampon[Title/Abstract])) OR (Menstrual Pad[Title/Abstract])) OR (Menstrual Pads[Title/Abstract])) OR (Pad, Menstrual[Title/Abstract])) OR (Pads, Menstrual[Title/Abstract])) OR (Menstrual Napkin[Title/Abstract])) OR (Menstrual Napkins[Title/Abstract])) OR (Napkin, Menstrual[Title/Abstract])) OR (Napkins, Menstrual[Title/Abstract])) OR (Feminine Napkins[Title/Abstract])) OR (Feminine Napkin[Title/Abstract])) OR (Napkin, Feminine[Title/Abstract])) OR (Napkins, Feminine[Title/Abstract])) AND (((((((((((((((((((((((((((((((((((((((((((((((((((((((Africa South of the Sahara[MeSH Terms]) OR (Africa South of the Sahara[Title/Abstract])) OR (Sub-Saharan Africa[Title/Abstract])) OR (Sub-Saharan Africa[Title/Abstract])) OR (Africa, Sub-Saharan[Title/Abstract])) OR (Africa South of the Sahara[Title/Abstract])) OR (Africa, Central[Title/Abstract])) OR (Cameroon[Title/Abstract])) OR (Central African Republic[Title/Abstract])) OR (Chad[Title/Abstract])) OR (Congo[Title/Abstract])) OR (Democratic Republic of the Congo[Title/Abstract])) OR (Equatorial Guinea[Title/Abstract])) OR (Gabon[Title/Abstract])) OR (Sao Tome[Title/Abstract] AND Principe[Title/Abstract])) OR (Africa, Eastern[Title/Abstract])) OR (Burundi[Title/Abstract])) OR (Djibouti[Title/Abstract])) OR (Eritrea[Title/Abstract])) OR (Ethiopia[Title/Abstract])) OR (Kenya[Title/Abstract])) OR (Rwanda[Title/Abstract])) OR (Somalia[Title/Abstract])) OR (South Sudan[Title/Abstract])) OR (Sudan[Title/Abstract])) OR (Tanzania[Title/Abstract])) OR (Uganda[Title/Abstract])) OR (Africa, Southern[Title/Abstract])) OR (Angola[Title/Abstract])) OR (Botswana[Title/Abstract])) OR (Eswatini[Title/Abstract])) OR (Lesotho[Title/Abstract])) OR (Malawi[Title/Abstract])) OR (Mozambique[Title/Abstract])) OR (Namibia[Title/Abstract])) OR (South Africa[Title/Abstract])) OR (Zambia[Title/Abstract])) OR (Zimbabwe[Title/Abstract])) OR (Africa, Western[Title/Abstract])) OR (Benin[Title/Abstract])) OR (Burkina Faso[Title/Abstract])) OR (Cabo Verde[Title/Abstract])) OR (Cote d'Ivoire[Title/Abstract])) OR (Gambia[Title/Abstract])) OR (Ghana[Title/Abstract])) OR (Guinea[Title/Abstract])) OR (Guinea-Bissau[Title/Abstract])) OR (Liberia[Title/Abstract])) OR (Mali[Title/Abstract])) OR (Mauritania[Title/Abstract])) OR (Niger[Title/Abstract])) OR (Nigeria[Title/Abstract])) OR (Senegal[Title/Abstract])) OR (Sierra Leone[Title/Abstract])) OR (Togo[Title/Abstract]))) AND ((((((((((((((((((((((((((Adolescent[MeSH Terms]) OR (Adolescent[Title/Abstract])) OR (Adolescents[Title/Abstract])) OR (Adolescence[Title/Abstract])) OR (Teens[Title/Abstract])) OR (Teen[Title/Abstract])) OR (Teenagers[Title/Abstract])) OR (Teenager[Title/Abstract])) OR (Youth[Title/Abstract])) OR (Youths[Title/Abstract])) OR (Adolescents, Female[Title/Abstract])) OR (Adolescent, Female[Title/Abstract])) OR (Female Adolescent[Title/Abstract])) OR (Female Adolescents[Title/Abstract])) OR (Adolescents, Male[Title/Abstract])) OR (Adolescent, Male[Title/Abstract])) OR (Male Adolescent[Title/Abstract])) OR (Male Adolescents[Title/Abstract])) OR (Young Adult[MeSH Terms])) OR (Young Adult[Title/Abstract])) OR (Adult, Young[Title/Abstract])) OR (Adults, Young[Title/Abstract])) OR (Young Adults[Title/Abstract])) OR (Child[MeSH Terms])) OR (Child[Title/Abstract])) OR (Children[Title/Abstract]))

Google Scholar

allintitle: "Menstrual hygiene practice" AND Adolescent

allintitle: "Menstrual hygiene management" AND Adolescent

allintitle: "Menstrual hygiene products"

CINAHL

(MH "Menstrual Cycle+") OR (MM "Menstruation") OR (MM "Menstrual Hygiene Products+")

African Journals online database

"Menstrual hygiene practice"

"Menstrual hygiene management" AND Adolescent
