# Supplementary material for: Menstrual hygiene practice and associated factors among adolescent girls in sub-Saharan Africa: a systematic review and meta-analysis
Source: BMC Public Health. 2023 Jan 6;23:33. doi: 10.1186/s12889-022-14942-8 (PMC9817285; doi:10.1186/s12889-022-14942-8)
Supplement: Supplementary file 3 — Additional file 3: Table S1. Meta-regression analysis of factors with menstrual hygiene practice in Sub-Saharan Africa, 2022 [file 12889_2022_14942_MOESM3_ESM.docx]

Additional file 3

Table S1: Meta-regression analysis of factors with menstrual hygiene practice in Sub-Saharan Africa, 2022

**
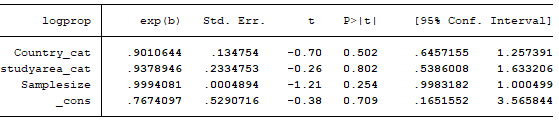
**
