# Supplementary figures and images for: Menstrual hygiene practice and associated factors among adolescent girls in sub-Saharan Africa: a systematic review and meta-analysis
Source: BMC Public Health. 2023 Jan 6;23:33. doi: 10.1186/s12889-022-14942-8 (PMC9817285; doi:10.1186/s12889-022-14942-8)

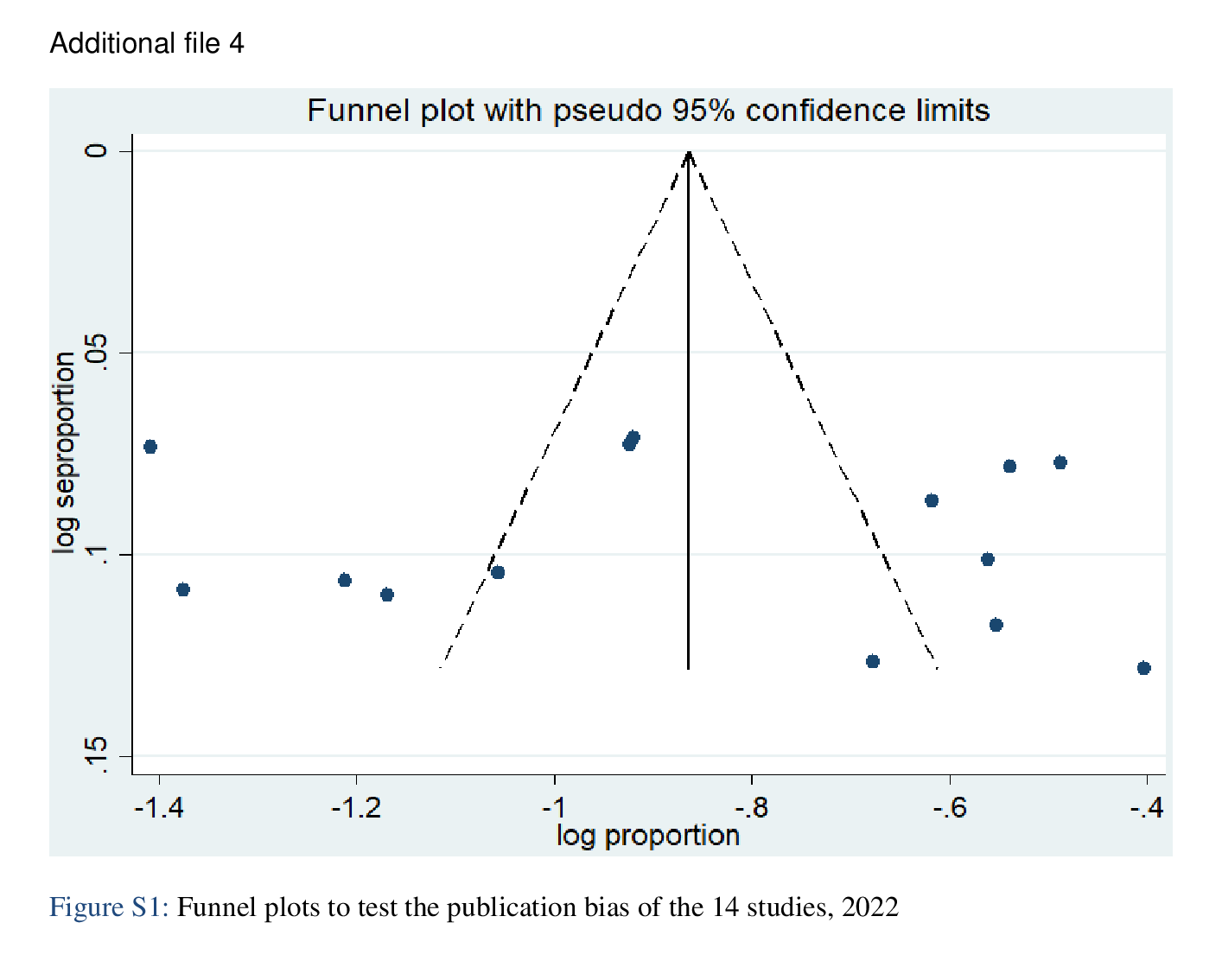

Supplement: Supplementary file 4 — Additional file 4: Figure S1 Funnel plots to test the publication bias of the 14 studies, 2022 [file 12889_2022_14942_MOESM4_ESM.tiff]
