# Supplementary material for: Menstrual hygiene practice and associated factors among adolescent girls in sub-Saharan Africa: a systematic review and meta-analysis
Source: BMC Public Health. 2023 Jan 6;23:33. doi: 10.1186/s12889-022-14942-8 (PMC9817285; doi:10.1186/s12889-022-14942-8)

Additional file 5

Table S2: Publication bias using Egger’s test on menstrual hygiene among adolescents in sub Saharan Africa, 2022


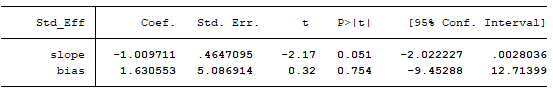

Supplement: Supplementary file 5 — Additional file 5: Table S2. Publication bias using Egger’s test on menstrual hygiene among adolescents in sub Saharan Africa, 2022 [file 12889_2022_14942_MOESM5_ESM.docx]
