# Supplementary material for: Menstrual hygiene practice and associated factors among adolescent girls in sub-Saharan Africa: a systematic review and meta-analysis
Source: BMC Public Health. 2023 Jan 6;23:33. doi: 10.1186/s12889-022-14942-8 (PMC9817285; doi:10.1186/s12889-022-14942-8)
Supplement: Supplementary file 6 — Additional file 6: Supplementary file Table 3. Quality appraisal of excluded studies [file 12889_2022_14942_MOESM6_ESM.docx]

Supplementary file Table 3: Quality appraisal of excluded studies

| Studies | Reason for exclusion |
| --- | --- |
| Ferahtia A. [68] | Age above inclusion criteria (> 19) |
| Abor PA. [69] | Age above inclusion criteria (> 19) |
| Hennegan J, et al. [70] | Intervention study |
| Korir E, et al.[71] | Age above inclusion criteria (> 19) |
| Akinwaare MO, et al.[72] | Outcome not reported |
| Garba I, et al.[73] | Age cut point not clearly defined |
| Aliyu I. et al. [74] | Outcome not reported |
| Ajaegbu Victoria U. et al. | Age above inclusion criteria (> 19) |
| Nabwera HM. et al.[75] | Age above inclusion criteria (> 19) |
| Magayane R, et al.[76] | Age above inclusion criteria (> 19) |
| Haftu Berhe [77]. | Age above inclusion criteria (> 19) |
| Shibeshi BY. et al. [78] | Age cut point not clearly defined |
| Ibaishwa RL, et al [79] | Age above inclusion criteria (> 19) |
| Shallo SA, et al.[80] | Age above inclusion criteria (> 19) |
| Crankshaw TL, et al. [81] | Age above inclusion criteria (> 19) |
